# Medical AI: are Osler's principles lost in translation?

**DOI:** 10.3389/fmed.2026.1991244

**Published:** 2026-10-22

**Authors:** Michel Goldman, Elie Cogan, Serge Goldman

**Affiliations:** Université Libre de Bruxelles, Brussels, Belgium

**Keywords:** AI, education, medicine, Osler, patient, physician, training

## Introduction

Artificial intelligence (AI) is poised to transform clinical practice, but its adoption coincides with a longstanding decline in bedside medicine. Herein, we argue that reintroducing Oslerian principles into medical training may be essential to ensure that technological progress does not come at the expense of the physician-patient relationship. Throughout, we use the term AI to refer to tools built on algorithms, from risk calculators and predictive models to large language models, that produce predictions, classifications or recommendations. These tools have no agency of their own: they operate only on the data supplied to them, and their implementation depends on how clinicians and institutions design and deploy them.

William Osler never saw an algorithm. He died in 1919, a century before “artificial intelligence” entered the clinical vocabulary. And yet no figure in the history of medicine is better equipped to tell us what we are about to lose (1). Osler's central achievement was a discipline of the mind: the conviction that the practice of medicine must rest on direct observation of the patient and on the relationship between patient and physician. We argue that this discipline is now under direct threat, and that AI tools, as currently deployed, tend to hasten its erosion rather than reverse it — unless we act deliberately to stop it. In fact, the physician-patient relationship has already been reshaped by decades of patient access to medical information through the internet and social media. This shift has been amplified by the development of health-related AI tools, which increase patients' knowledge but also expose them to misinformation and misinterpretation. Nowadays, patients often arrive at the clinical encounter having already formed their own interpretations about their disease, which directly impacts the dialogue with the physician from the very first minutes of the encounter — a dynamic Osler never had to confront. Oslerian principles must therefore be adapted to this new reality, in which both physician and patient increasingly approach the clinical encounter already shaped by algorithms.

## Clarifying what AI can do — and will not do in the foreseeable future

AI tools can go extraordinarily far in the medical process — synthesizing literature at a scale no human can match, flagging patterns in imaging and pathology that trained eyes miss, drafting notes, triaging risk, generating differential diagnoses, and suggesting therapeutic options in seconds. None of this is in serious dispute anymore, and clinicians who dismiss it outright are being unrealistic.

On the other hand, it is illusory to imagine that AI will, in the foreseeable future, replace clinical observation and the personal relationship between doctor and patient. This needs to be said plainly, not hedged. Diagnosis by pattern-matching on data is not the same as sitting at the bedside of anxious patients, watching how they behave, picking up on what they leave unsaid, and actually building the trust that makes adherence possible. Whatever level of performance AI models will achieve in the future, they will remain trained on the past, and no model can substitute for what happens, uniquely, in the present, between two people.

There is also a more technical reason why bedside medicine remains indispensable: AI can only reason about the reality that has been captured and digitized. An algorithm implicitly assumes that what is absent from the record does not exist. Because important information reaches the record only if a physician observes it, an algorithm working from an incomplete record can return a seemingly precise but inaccurate output, creating an illusion of certainty. Oslerian observation is therefore not a nostalgic complement to AI but a technical prerequisite for its validity: no AI tool can compensate for a hurried or incomplete examination. Far from making clinical skills obsolete, AI demands sharper skills in listening, observation and physical examination.

## A trend already underway — and poised to worsen

Losing the lessons learned at the bedside is not a hypothetical risk, nor is it a new one. For decades, medical departments have been organized around organ systems and specialized technologies. This process of specialization and subspecialization — a transformation that long predates the current wave of AI adoption — has produced remarkable diagnostic and therapeutic advances, but it has also fragmented the physician's view of the patient as a whole and made a holistic approach harder to sustain. Another powerful driver of this change has been the growth of administrative and documentation burden: in ambulatory practice, a time-and-motion study found that physicians spend nearly two hours on electronic health records and desk work for every hour of direct clinical face time with patients (2), and administrative burden has been associated with lower intrinsic motivation among physicians and poorer patient-reported experience of care (3).

Consistent with this, studies among internal medicine trainees have documented a clear decline in direct time with patients for well over a decade (4), and the trend is still underway today in several disciplines. For example, in hematologic malignancies, where AI-based tools are being rapidly adopted to guide diagnosis, prognosis, and treatment decisions (5), physicians increasingly spend more time in offices in front of computer screens — reviewing algorithmic outputs, multiomic profiles, and risk calculators — often making therapeutic decisions before they examine the patient, so that the consultation risks becoming a confirmation of a decision already made elsewhere, rather than the encounter from which the decision should emerge.

Deployed carelessly, AI tools are likely to deepen this pattern rather than correct it. Many current incentives push in that direction: institutions will reward the efficiency of deciding early, physicians will gravitate toward the workflow that is faster and less cognitively taxing, and the tools themselves are easiest to deploy on data that are already structured and available before the visit. Left to market and workflow pressures alone, AI is unlikely to restore the clinical encounter and may further hollow it out. This is the central danger. However, AI can also work in the opposite direction. In a recent randomized trial, ambient AI scribes, which transcribe the consultation and draft the clinical note, were associated with reduced physician burnout, and doctors reported that the technology allowed them to engage better with their patients (6). Such tools can return the physician's attention from the screen to the patient — provided that the time they free is deliberately protected for the clinical encounter.

## Osler's principles are the countermeasure

This is precisely why Osler's teaching should not be treated as an outdated precept, but as the specific countermeasure this moment requires. We argue that it should be urgently reintroduced as a structural component of clinical education, integrated into, not appended to, the technical curriculum. Three Oslerian principles should anchor that effort:

“Listen to the patient; he is telling you the diagnosis.” What the physician personally learns at the patient's bedside should always come first — a principle physicians in subspecialty training are especially at risk of losing, as AI output can easily become the main input they consult before reaching a conclusion. AI output should inform that conclusion but not replace it.

“Medicine is learned by the bedside and not in the classroom.” Osler's most consequential reform was moving medical education out of the lecture hall and to the patient's side. A century later, the direction of travel has reversed: trainees now often learn to interpret data before they learn to examine a patient. That sequence needs to be corrected, with protected, unhurried bedside time built into training and explicitly shielded from the pressure to consult the algorithm first.

“Imperturbability.” In his famous 1889 address, Aequanimitas (7), Osler used this word to describe the calm and steady judgment a physician needs when facing suffering, urgency and clinical uncertainty. Applied to the output of AI, the same discipline must hold: neither adopting it blindly, a failure known as automation bias (8), nor dismissing it out of hand, but checking it as rigorously as any other clinical evidence, including against what the physician has personally observed.

These principles cannot be reintroduced as a simple return to an earlier era; they must be adapted to a clinical environment now shaped not only by AI but also by electronic health records, an ever-expanding volume of diagnostic data, and patients arriving informed — and sometimes misinformed. The task for training programs is not to teach learners to resist these developments but to integrate them without displacing direct observation and reasoning. Concretely, this could include bedside practice that requires trainees to form their own clinical impression before consulting AI output, case studies comparing bedside findings with AI-generated information, exercises showing how omissions in the history or examination alter an algorithm's output, and communication exercises on discussing AI-derived information with patients. These skills should be embedded into formal assessment and not treated as incidental.

## Concluding remarks

Discussions on “AI and the future of medicine” too often lead to wishful thinking that is very unlikely to be implemented. Instead, they should lead to concrete proposals, such as treating the clinical encounter as a parameter to be measured in academic medical centers. Time at the bedside is a necessary but insufficient indicator, since more time does not automatically translate into better care. We therefore propose mixed metrics combining the time physicians actually spend with patients, the diagnostic accuracy of the physician working with AI compared with either alone, and patients' perceptions of empathy and trust assessed with validated instruments such as the Consultation and Relational Empathy (CARE) measure (9).

William Osler would have welcomed the advent of AI in medicine, but he would have insisted that every AI tool be held to a single non-negotiable question: does it bring the physician closer to the patient, or does it interpose itself between them? On present evidence, in disciplines already reorganized around screens and algorithms, many AI deployments are failing that test (10). The remedy is not to slow the technology down. It is to speed up, urgently, the reintroduction of Osler's principles into the training of every physician who will use it.
